# NGS data vectorization, clustering, and finding key codons in SARS-CoV-2 variations

**DOI:** 10.1186/s12859-022-04718-7

**Published:** 2022-05-17

**Authors:** Juhyeon Kim, Saeyeon Cheon, Insung Ahn

**Affiliations:** 1 Department of Data-Centric Problem Solving Research, Korea Institute of Science and Technology Information, Yuseong-gu, Daejeon, Korea; 2 Center for Convergent Research of Emerging Virus Infection, Korea Research Institute of Chemical Technology, Yuseong-gu, Daejeon, Korea; 3 Applied Artificial Intelligence Major, University of Science & Technology, Yuseong-gu, Daejeon, Korea; 4 Department of Industrial Engineering, Ajou University, Suwon, South Korea

**Keywords:** SARS-CoV-2, Protein sequence analysis, Sequence data pre-process, t-Stochastic neighbour embedding, Density based spatial clustering of applications with noise, Clustering, Random forest, Shapely value, Feature selection

## Abstract

The rapid global spread and dissemination of SARS-CoV-2 has provided the virus with numerous opportunities to develop several variants. Thus, it is critical to determine the degree of the variations and in which part of the virus those variations occurred. Therefore, in this study, methods that could be used to vectorize the sequence data, perform clustering analysis, and visualize the results were proposed using machine learning methods. To conduct this study, a total of 224,073 cases of SARS-CoV-2 sequence data were collected through NCBI and GISAID, and the data were visualized using dimensionality reduction and clustering analysis models such as T-SNE and DBSCAN. The SARS-CoV-2 virus, which was first detected, was distinguished from different variations, including Omicron and Delta, in the cluster results. Furthermore, it was possible to examine which codon changes in the spike protein caused the variants to be distinguished using feature importance extraction models such as Random Forest or Shapely Value. The proposed method has the advantage of being able to analyse and visualize a large amount of data at once compared to the existing tree-based sequence data analysis. The proposed method was able to identify and visualize significant changes between the SARS-CoV-2 virus, which was first detected in Wuhan, China, in December 2019, and the newly formed mutant virus group. As a result of clustering analysis using sequence data, it was possible to confirm the formation of clusters among various variants in a two-dimensional graph, and by extracting the importance of variables, it was possible to confirm which codon changes played a major role in distinguishing variants. Furthermore, since the proposed method can handle a variety of data sequences, it can be used for all kinds of diseases, including influenza and SARS-CoV-2. Therefore, the proposed method has the potential to become widely used for the effective analysis of disease variations.

**Supplementary Information:**

The online version contains supplementary material available at 10.1186/s12859-022-04718-7.

## Introduction

Just before entering into the new year 2020, a group of unknown pneumonia patients in Wuhan, Hubei province, China was confirmed to be infected with a novel coronavirus, Severe Acute Respiratory Syndrome Corona Virus 2 (SARS-CoV-2) [1]. The first whole genome sequence of the SARS-CoV-2 virus was released by the National Centre for Biotechnology Information (NCBI) Genbank on January 5, 2020 [2]. The first human-to-human transmission of this virus was confirmed on January 14, 2020; however, the virus had already spread to many countries around the world by that time. As the situation quickly worsened, the World Health Organization (WHO) declared a SARS-CoV-2 pandemic on March 11, 2020.

Since the start of the pandemic, various mutations in SARS-CoV-2 have been observed, and more variations continue to emerge [2, 3]. The rapid global spread and dissemination of SARS-CoV-2 has given the virus numerous opportunities to mutate. In particular, mutations such as D614G in its spike protein enhanced the viability of the virus [4, 5]. It is therefore critical to determine the level of SARS-CoV-2 mutation and in which part of the virus those mutations have occurred. Therefore, many researchers are trying to analyse coronaviruses from various perspectives and approaches in order to track newly emerging variants and determine their characteristics. Studies reported that mutations may occur according to geographic location by comparing viral sequences collected from Asia, Africa, Europe, North America, South America and Oceania with the SARS-CoV-2 virus that was First emerged in Wuhan, China in December 2019 [6, 7]. The mutations in various parts of the spike protein are known to affect areas such as infectivity, disease severity, or interaction with the host [8, 9]. Furthermore, studies showed that mutations in the virus may affect the effectiveness of vaccines [10, 11]. Consequently, many studies argued that it is of paramount importance to continuously monitor and conduct research on changes that occur in the virus.

Next Generation Sequencing (NGS) based characterization has contributed to increase insight into SARS-CoV-2 genome organization and transcriptional complexity [12, 13]. One of the most used methods to track mutations of SARS-CoV-2 through these NGS-based data is by analysing the phylogenetic tree. Phylogenetic analysis forms evolutionary relationships or trees, and traces their evolution. Since there was insufficient data at the beginning of the SARS-CoV-2 epidemic, a phylogenetic tree was used to investigate the mutation of the virus [14–16]. Phylogenetic analysis is also used in the field of discovering effective therapeutic candidates for viruses [17]. Morel, B. et al. proposed a numerical mapping method using machine learning to predict the protein interaction between SARS-CoV-2 and humans, which is an important factor in understanding the biological activity of organisms [18]. Furthermore, phylogenetic analysis and geographic location were used to determine the specificity of SARS-CoV-2, and research based on this data are currently underway [6, 7, 19–22]. However, Khan, A. et al. argued that it was not easy to infer phylogeny using large amounts of data, as variants of SARS-CoV-2 appeared over time and there were too many sequence data for a few variants [16]. As a result, unless a large budget and manpower were invested, analysing a large amount of data at once and visualizing it in the analysis using existing traditional sequencing data was difficult. Even if new data was analysed, it was difficult to distinguish whether the new virus was a mainstream virus or a non-mainstream virus. Therefore, a methodology that can perform analysis together by maximizing the overflowing SARS-CoV-2 sequencing data is required. By analysing a large amount of data together, it was possible to analyse the data more objectively and find out how many viruses are in a group and how many viruses are occurring in a specific period, where in the world they are occurring, and what differences exist between the variant groups.

In this study, different methods were proposed that can be used to vectorize the SARS-CoV-2 spike protein sequence data collected from the NCBI Genbank and Global Initiative for Sharing All Influenza Data (GISAID), perform clustering analysis, and visualize the results. After pre-processing a total of 224,073 SARS-CoV-2 sequencing data using the method proposed, it was possible to verify that various mutations were clustered by several characteristics. In addition, it was possible to visualize this as a two-dimensional graph and display the results in a table classified into various indicators for statistical analysis. The proposed methodology also allowed researchers to examine mutant viruses and determine which codons of the spike protein were modified before investigating those codons. The rest of this paper is organized as follows: The Methods section outlines which data was used to test the proposed method and describes the methods that were used to pre-process and vectorize the data. The section also explains t-Stochastic Neighbour Embedding (t-SNE), a dimensionality reduction technique used to visualize data, and briefly introduces the Density Based Spatial Clustering of Applications with Noise (DBSCAN) techniques used to cluster the pre-processed data. Finally, the random forest technique used to find the codons in which the virus mutation occurred was described. In the Experiment section, the parameter settings used for the machine learning methods (i.e., the t-SNE, DBSCAN, and random forest methods) were briefly explained and discussed how the data was structured. Next, the experimental findings are briefly described in the Results section before finally presenting the Discussion and Conclusion sections.

## Methods

Since the spike proteins in SARS-CoV-2 are known to play a key role in mediating infection in human cells, this study proposed a method for vectorizing and clustering the spike protein sequence data from SARS-CoV-22 [6–10, 21]. In the following Data section, the pre-processing method for vectorizing the data was described, and the t-SNE and DBSCAN methods used for clustering were briefly explained, as was virus distribution visualization based on the pre-processed data. Classical methods such as Principal Component Analysis (PCA), Independent Component Analysis (ICA), and Multi-Dimension Scaling (MDS) are methods used for reducing the dimension of data. However, these classic dimensionality reduction techniques were not suitable for handling very large amounts of data and very high dimensionality. Since then, dimensionality reduction techniques such as Locally Linear Embedding (LLE) and Isometric Feature Mapping (ISOMAP) were proposed. The LLE and ISOMAP are methods of learning a low-dimensional space that preserve the structure of a high-dimensional space using nearest neighbours’ information. However, LLE and ISOMAP are suitable for visualizing a data space where manifolds exist, such as swissroll data, rather than visualizing the embedding space of deep learning models. Therefore, these methods were not suitable for visualization of high-dimensional data because they did not preserve the information required for visualization. Recently, methods such as t-SNE and Autoencoder are the most widely used methods. The t-SNE works well for data with different distributions for each feature because it captures the distribution-based hidden factor of high-dimensional features very well. Furthermore, as it is not very sensitive to parameter setting, it is a technique suitable for non-professionals to use. Finally, Autoencoder is one of the latest dimensionality reduction techniques based on deep learning. However, Autoencoder has a problem in which performance changes significantly depending on how layers are added or parameters are adjusted. As a result, unless you are an expert in the field, there are significant limitations associated with this technique. For these reasons, in this study, t-SNE, which can be used by anyone and has good performance, was adopted as the dimensionality reduction method. To judge the performance of various dimensionality reduction techniques, results from MDS, LLE, ISOMAP, t-SNE, and Autoencoder were added using sample data from the collected data in Additional file 1. Finally, after the data have been clustered, the Random Forest technique was used to identify the part of the sequence data which played a big role in dividing the cluster. Features that played a major role in clustering were explored because significant mutations were expected to have occurred in those features. Next, we briefly described the sources from which the data used in this study were collected, what types of data were used, and how the data were pre-processed. In addition, brief description how and for what purpose the machine learning techniques utilized were used.

### Data

The SARS-CoV-2 spike protein sequences were collected from two open-source databases, NCBI’s SARS-CoV-2 Data Hub and GISAID [23, 24]. The SARS-CoV-2 sequencing data provided by NCBI and GISAID was collected and used because the sequencing data provided by both databases is the most used in SARS-CoV-2 research worldwide. After SARS-CoV-2 was discovered, sequencing data of this virus was published for the first time in the GenBank of the NCBI [25]. In the early days of the virus spread, a lot of SARS-CoV-2 sequencing data was provided through the GenBank, and many SARS-CoV-2 related studies were conducted using the sequencing data collected from the GenBank [26–30]. Therefore, in this study, data provided from NCBI's GenBank was used as the initial data. Since then, GISAID has been provided by tagging the type of mutation in sequencing data after various mutated viruses have occurred and started to spread. The GISAID database is also being actively used in various recent SARS-CoV-2 related studies [7, 19–22]. Since the NCBI database provides the SARS-CoV-2 virus for each gene, the nucleotide sequences encoding spike proteins were selected and downloaded (collection period: Dec. 2019–Jan. 2021). The mutant virus information about SARS-CoV-2 was also downloaded from GISAID’s newly established EpiCoV™ platform and used in the whole genome form. Since mutant virus data were not provided for each gene, each whole genome was extracted and used after matching the site of the gene on the whole genome through alignment with the spike protein of the virus first discovered in Wuhan, China (accession number: MT019529.1, protein ID: QHU36824.1). The NCBI GenBank’s accession number MT019529.1 SARS-CoV-2 sequencing data is one of the first virus sequencing data collected in Wuhan, China, and is one of the several SARS-CoV-2 reference viruses. In many studies, sequencing data alignment was performed using MT019529.1 data, one of the reference data, so this study also performed sequencing data alignment using the data [20, 31–33]. Protein ID QHU36824.1 is an ID indicating the part corresponding to spike Protein among the entire SARS-CoV-2 sequencing data. A spike protein corresponding to protein ID QHU36824.1 was analysed in various SARS-CoV-2 studies, and since this study also aimed to analyse mutations in the spike protein, the portion corresponding to protein ID QHU36824.1 in the overall sequencing data was extracted and used [34–37]. Variant virus data were obtained from six strains, namely B.1.1.7 (Alpha), B.1.351 (Beta), P.1 (Gamma), B.1.617 (Delta), B.1.1.529 (Omicron), B.1.640 and some unlabelled data were collected. A maximum of 5,000 sequences were used per month to avoid overfitting the data, and not more than 10 sequences were extracted per collection day to avoid virus clustering on a specific day.

Annotation information was categorized by country and area, year, month, and date of collection to analyze the sequences. The NCBI provides sequence data that are divided into different proteins, including ORF, nucleocapsid, spike, or envelope proteins. Spike proteins mediate infections in human cells, and they are the targets of most vaccine strategies and antibody-based therapeutic approaches [38, 39]. Continent, country, year, and month information was tagged together in the collected data. The data used in the present study were data collected by NCBI from December 2019, when this corona virus was first discovered in Wuhan, China, to February 2021, and data collected by GISAID from January 2020 to December 2021. By region, the following number of data samples were collected: Data was collected and analysed from six continents: Asia, Africa, North America, South America, and Oceania, with the exception of Antarctica, where no confirmed cases have occurred. The reference virus was discovered in Wuhan, China in 2019, and mutations such as Alpha, Beta, and Gamma were identified in the second half of 2020, Delta mutations in the first half of 2021, Omicron mutations, and 490R-GH mutations were discovered in the second half of 2021. Regarding the collected data, a detailed table of data collected by each continent and a detailed table of data collected by variant were added to the Additional file 1.

**Fig. 1:**
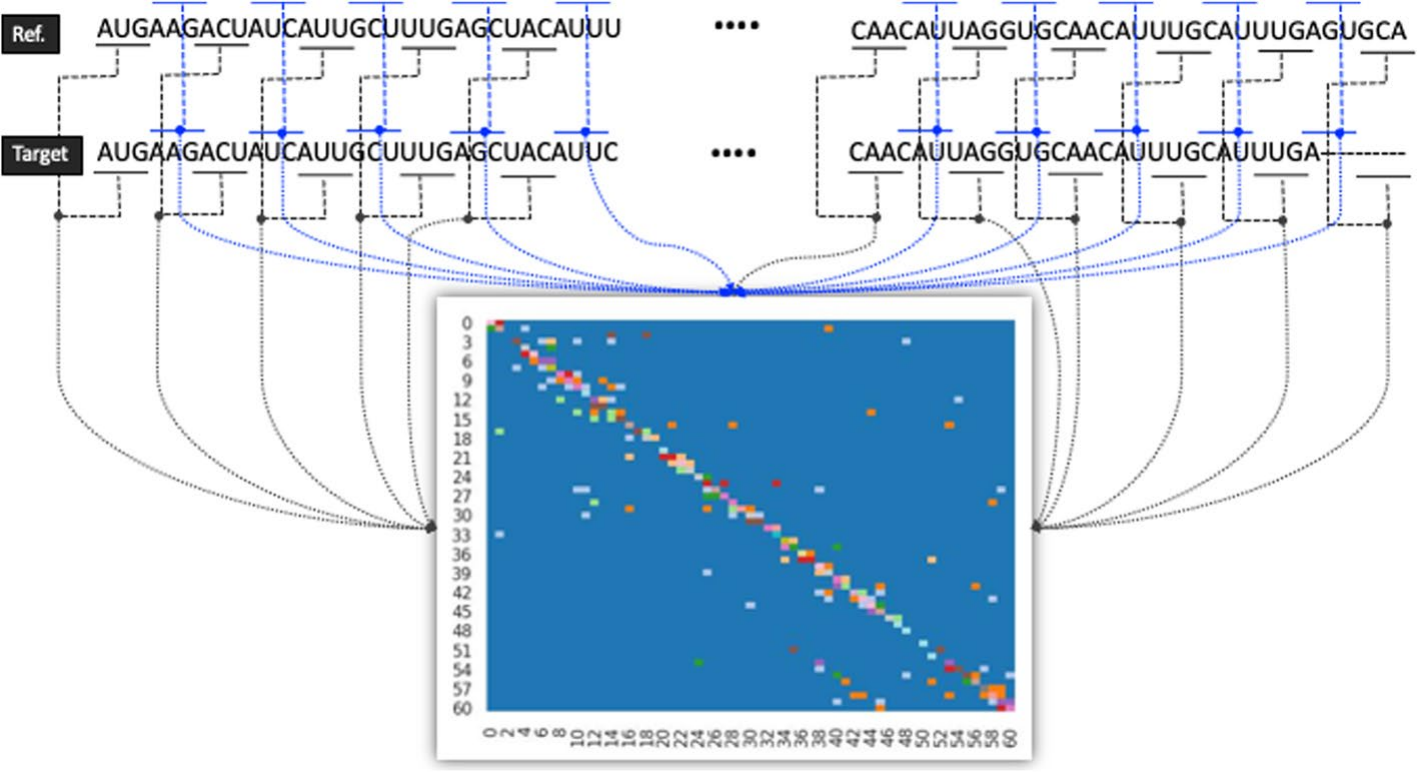
Process of generating a 61 × 61 variation matrix through comparative analysis of the aligned reference and target sequences at the codon level

In this analysis, each virus was represented by one variation vector, and these vectors were then used for the clustering analysis, which is a differentiating method from the other analyses. The most pertinent issue in SARS-CoV-2 research is whether mutations have happened since the original virus was detected, and if so, what these mutations look like. In this study, the spike protein of the virus first discovered in Wuhan, China (accession number: MT019529.1, protein ID: QHU36824.1) was set as the reference sequence, and any virus sequence collected later was compared with this reference sequence to vectorize any mutation progress. In detail, the method for generating a variation vector proceeds as follows:

First, multiple sequence alignment (MSA) was performed between the reference sequence and each target sequence using the mafft program [40]. Two aligned sequences with the same length (3,822 bp), including gaps as a result of MSA, were sequentially divided into individual codon units, each of which consist of three bases, and then any variations between the reference and target sequence from the beginning of the sequence to the end were counted. Based on these counting results, a 61 × 61 matrix was generated, which is the total number of codons encoding amino acids in both rows and columns (Fig. 1).

To use the collected data as input for machine learning processing, pre-processing was needed. First, a method for vectorizing the sequence data was proposed. To vectorize the sequence data, a piece of reference data must first be selected, then each collected piece of data was compared to the reference data to identify any differences. For example, as shown in Fig. 1, the similarity by codon between the reference data and target data can be expressed as a heatmap. In the heatmap, blue dots refer to 0 similarities while other dots mean a similarity of 0 to 1. The similarity by codon can also be expressed as a matrix. As each data sequence consisted of 61 values, the pre-processed data was in the form of a 61 by 61 matrix, so one set of comparative data was composed of 3,721 attributes (61 × 61 = 3,721). In this study, one of the SARS-CoV-2 virus cases that occurred in Wuhan, China in January of 2020 (accession number: MT019529.1, protein ID: QHU36824.1) was selected as the reference data. Then, all the accumulated data used in this analysis was pre-processed into a form in which it was usable for our experiments according to the proposed method. A custom python code was written to pre- and post-process all the sequence data used in this study, and the MySQL Database Management System was used to effectively manage the collected data.

### t-SNE

Since a single sample in our experiments consisted of 3,721 attributes, the t-SNE technique was used to compress all samples into two-dimensional data to visualize and display the data. Various visualization methods can be used to help understand high-dimensional data. Since humans are familiar with and understand two- and three-dimensional space, it was necessary to reduce the dimensions of high-dimensional data while preserving all similarities between points in the original data space so that two vectors that were similar in high-dimensional space still appear similar when represented in two-dimensional space. One of the most effective dimension reduction methods was the t-SNE technique. t-SNE is used to expresses high-dimensional data low-dimensional space by finding a low-dimensional embedding vector that preserved the neighbour structure in the high-dimensional data. t-SNE can achieve more stable embedding results than other dimension reduction algorithms for vector visualization because t-SNE converts the distance between pieces of data into stochastic probabilities and uses these for the embedding [41].

### DBSCAN

After the data dimensions have been reduced using t-SNE, each sample was labelled using DBSCAN to see how the data was clustered. The DBSCAN is a density-based clustering approach that clusters data by assuming that "similar data will be distributed close to each other" [42]. DBSCAN is a clustering algorithm that does not specify the number of clusters. Dense areas, which are called dense regions of data, were considered to constitute a cluster, while relatively empty areas were considered to be boundaries separating clusters. In simple terms, if there are more than *m* points within a radius *e* of a point, then this area is recognized as a cluster. These *m* and *e* are parameters to be set when using DBSCAN. In the beginning stages of DBSCAN, the target number of clusters does not need to be determined, allowing for nonlinear border clustering with the added benefit of noise resistance.

### Random forest

The importance of the features in the data was calculated using the random forest technique; specifically, this technique was used to predict what part of the virus mutated and formed different clusters. The random forest model is an ensemble machine learning model that forms several decision trees and passes new data points through each tree at the same time, then votes using the classification results from each tree, and the result with the most votes is given as the final classification [43]. Some trees generated by the random forest method can become overfitted; however, by creating numerous trees, occasional results that suffer from overfitting do not significantly affect the final prediction result. The random forest method measured the importance of a feature based on how much it contributes to improving accuracy and node impurity [43]. This allows us to extract the parts of the data that play an important role.

## Experiments

### Experimental settings

For this experiment, a total of 224,073 SARS-CoV-2 spike protein sequences were collected from various countries around the world over the period from December 2019 to December 2021. This data was pre-processed using one virus found in Wuhan, China in January 2020 as the reference sequence. Each data entry consisted of 3,721 attributes, and the dimensions of the data were reduced using t-SNE to visualize the data. Then, the data was clustered and labelled using DBSCAN. Through the t-SNE data dimension reduction technique, the dimensions of our data were reduced to two, and a value of 3,000 was used as the learning rate parameter. For the DBSCAN clustering technique, the eps and minimum sample parameters were chosen as 2 and 10, respectively, as these values produced the best clustering results. In the selection of variable importance for the random forest classifier, 80% of the data was used as training data and the remaining 20% was used as validation data to see whether the model actually classified each cluster well. The verification results showed that the variables selected by the random forest technique were important variables. If the proposed method can show how viruses form significant clusters, this will make it possible to directly check how active variations of SARS-CoV-2 have progressed. Further, the country as well as occurrence month and year information tagged in the data can be used to determine when and where these variations occurred. The collected data cover samples from a total of 125 different countries, with 56,139 data points from the United Kingdom, accounting for 25% of the total, and the second-most observations coming from the U.S., making up about 19% of the total. Countries such as France, Mexico, Australia, and India followed in terms of the number of samples provided.

### Clustering analysis results

**Fig. 2:**
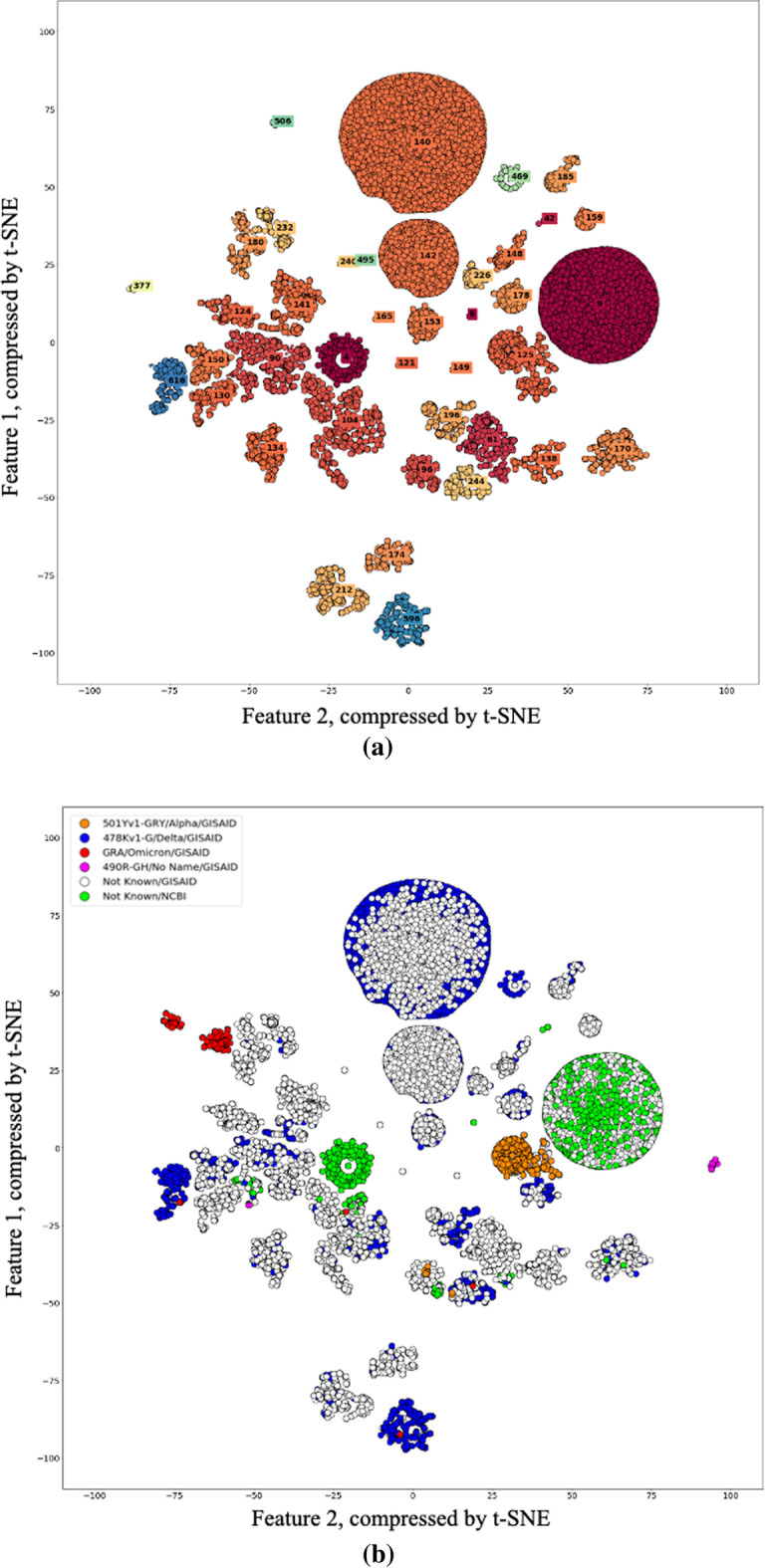
Results of clustering using Density Based Spatial Clustering of Application with Noise (DBSCAN): **a** shows clusters containing over 1,120 data points, and **b** shows the types of variants tagged in each virus by colour: In (**a**), it can be seen that numerous viruses gather to form a specific cluster, and in (**b**), it can be seen that one cluster tends to consist of a specific mutant virus

After using t-SNE to reduce the dimensions of the pre-processed data into two, DBSCAN was used to cluster the 224,073 samples into 672 different clusters, as shown in Fig. 2. Next, clusters containing more than 1,120 samples, which were approximately 0.5% of the clusters, were extracted to eliminate relatively small groups and identify thicker stems. However, among the small groups, the clusters containing the new variants Omicron and B.1.640 were added and displayed. In total, 41 different clusters were extracted in this way, and 124,387 samples were included in these extracted clusters. This means that over 55.5% of the total data was included in the top 23 clusters, which, again, only account for 6.1% of all clusters. The proposed method confirmed that different variants form a cluster when the colour corresponding to each variant in Fig. 2a was applied using GISAID data that gave variant information combined. Moreover, despite the fact that large amounts of data were clustered without labels, the data formed many distinct clusters, thus confirming that numerous variations have already occurred in the spike proteins. It was also confirmed that only a small number of clusters became widespread. To examine the characteristics of each cluster, information such as where and when the virus occurred was organized and examined.

**Fig. 3:**
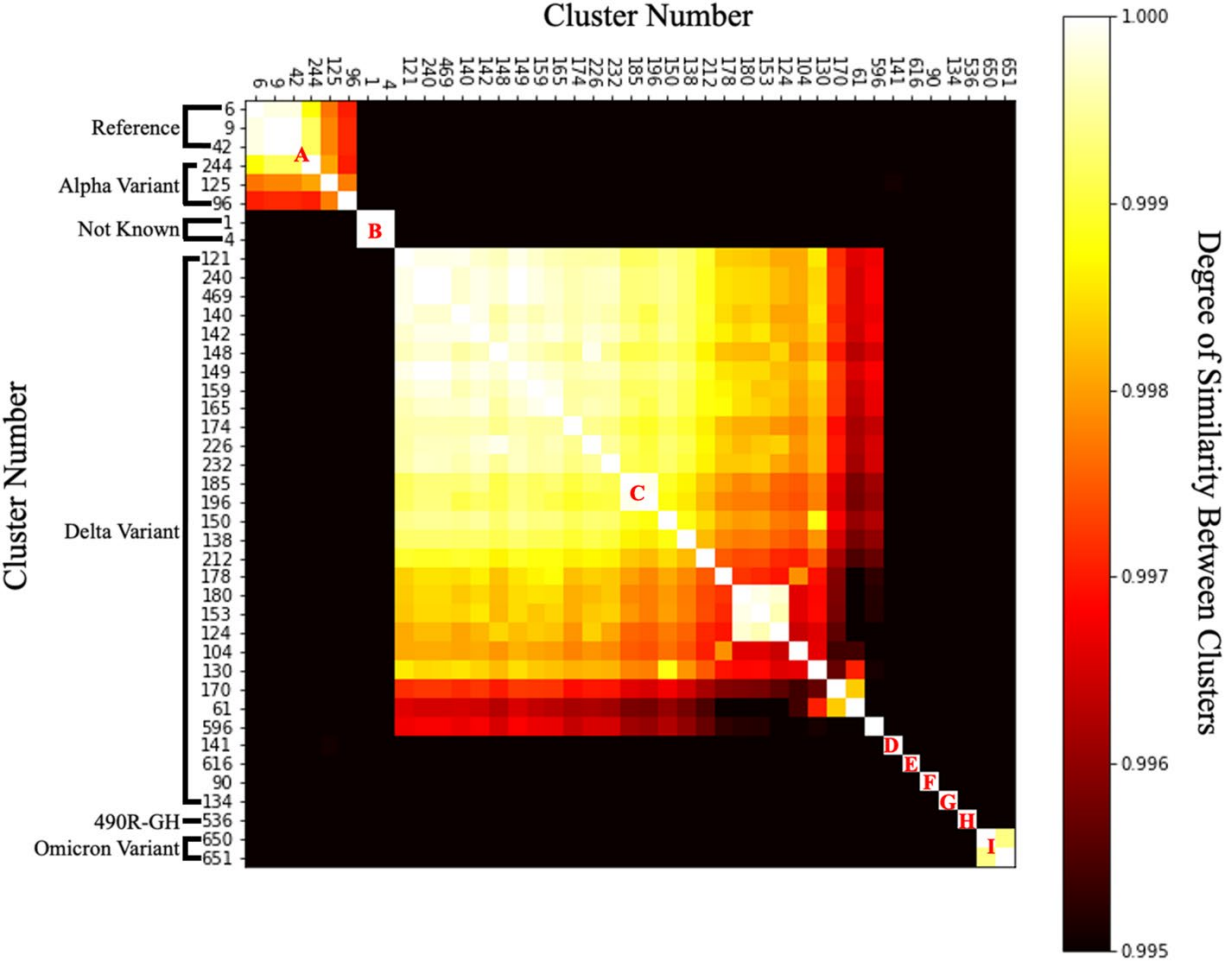
A heatmap showing the similarity between each virus cluster and clusters similar to each other in groups A to I (the brighter the more similar; the darker the more dissimilar): Clusters 6, 9, and 42 with high similarity within group A were confirmed to be the reference virus first discovered in Wuhan, China in December 2019, 125 with low similarity is the Alpha variant called the British variant, viruses of group B are viruses with no variant information discovered in Oceania since mid-2020, viruses of groups C, D, E, F, G are Delta variant, viruses of group H are 490R-GH, and finally, group I of the virus was identified as an Omicron variant

Table 1 shows the types of viruses that make up clusters consisting of more than 1,120 data. Viruses that were labelled as “Not Known”, indicated that the viruses did not have virus mutation information. Therefore, these viruses could be the reference viruses first discovered in December 2019, or they could be the Alpha, Beta, Delta, or Omicron mutations. By examining at the time when the virus was identified or by searching at viruses that bind together in the same cluster, it was possible to figure out what kind of mutation these "Not Known" viruses were. Viruses in cluster 6, which consisted only of “Not Known” viruses, were the only group that contained viruses discovered in 2019, and could be considered as reference viruses. Groups 61, 121, 130, 134, 138, 140, etc., can all be considered as Delta variants because the only Delta variant was bound with the “Not Known” virus. In addition, it can be seen that cluster 536 was a recently discovered mutation, and clusters 650 and 651 were Omicron mutations. It can be seen that most of the clusters except for some clusters such as 9 and 104 were grouped with data tagged with a specific variant. Although several mutant viruses were included in groups 9 and 104, they could be regarded as mis-tagged because the number of specific mutations among them was very small. The credibility of the above interpretations was strengthened by the results shown in Fig. 3, which showed the similarity between each cluster. Figure 3 is a similarity heat map between clusters drawn by arranging clusters with high similarity to each other. Viruses formed nine clusters according to their similarity, and each cluster was given a name from A to I. Table 2 shows how many viruses belonging to each group occurred each quarter. Viruses from group A, including cluster 6, which were first discovered in December 2019 and peaked in the second quarter of 2020, whereas viruses from group B began to be discovered in the second quarter of 2020 and peaked in the third quarter. Next, in the case of cluster 125 including the alpha mutation first discovered in the UK, as shown in Fig. 2, although the alpha and delta mutations showed distinct clusters, they were organized into the same cluster. Therefore, in cluster 125, only the data tagged with the alpha mutation were separately extracted and the experiment was conducted. Then, it can be seen that viruses belonging to groups C, D, E, F, and G, including clusters of viruses currently classified as Delta variants, were discovered little by little in the first quarter of 2021, after which the number exploded in the second quarter. Interestingly, the virus tagged with the 'Delta' variants was divided into several clusters and groups. In response, we examined whether there are differences causing the variants classified similarly as 'Delta' to be divided into several different clusters and groups, and further investigate whether the viruses belonging to different groups than the first discovered virus group were caused by changes in any particular part of the virus. An experiment was conducted to see if it was divided into several clusters and groups. Subsequently, it can be seen that the 490R-GH mutation and the Omicron mutation, which began to be discovered in the fourth quarter of 2021, form different clusters. Each of the variants defined so far was classified due to mutations in a specific part of the virus, and the appearance of groups according to variants in the figure proved that the proposed model classified mutant viruses well. Furthermore, even though the virus is classified as a variant of the same kind due to a certain large characteristic change, subtypes due to other minor mutations may occur. A variant called Delta Plus appeared as a new branch from the Delta variant, and 656 and 269 mutations were observed, respectively [44]. Therefore, it is very important to identify and analyse subtypes due to small changes in the same variant group as shown in the results of Fig. 3.

**Table 1:** Number of viruses included in each label and degree of composition compared to total virus to be analysed, type and distribution of variants included in each label, and number of viruses included in the label by year

Labels	Variant3	Year				Total
		2,019	2,020	2,021	Count	
1	Not Known/NCBI	–	2,715	–	2,715	2,715
4	Not Known/NCBI	–	1,908	1	1,909	1,909
6	Not Known/GISAID	–	141	5	146	2,229
	Not Known/NCBI	12	2,071	–	2,083	
9	Delta	–	–	1	1	16,896
	Not Known/GISAID	–	1,581	892	2,473	
	Not Known/NCBI	–	14,202	220	14,422	
42	Not Known/GISAID	–	9	3	12	1,346
	Not Known/NCBI	–	1,325	9	1,334	
61	Delta	–	–	19	19	2,550
	Not Known/GISAID	–	4	2,288	2,292	
	Not Known/NCBI	–	233	6	239	
90	490R-GH	–	–	1	1	3,886
	Delta	–	2	1,162	1,164	
	Not Known/GISAID	–	28	2,266	2,294	
	Not Known/NCBI	–	404	23	427	
96	Alpha	–	–	10	10	1,147
	Delta	–	–	18	18	
	Not Known/GISAID	–	30	962	992	
	Not Known/NCBI	–	120	7	127	
104	Delta	–	–	769	769	5,024
	Not Known/GISAID	–	165	3,766	3,931	
	Not Known/NCBI	–	276	46	322	
	Omicron	–	–	2	2	
121	Delta	–	–	569	569	3,034
	Not Known/GISAID	–	–	2,465	2,465	
124	Delta	–	–	34	34	1,306
	Not Known/GISAID	–	–	1,272	1,272	
125	Alpha	–	861	1,370	2,231	4,447
	Delta	–	–	476	476	
	Not Known/GISAID	–	14	1,620	1,634	
	Not Known/NCBI	–	48	58	106	
130	Delta	–	–	21	21	1,260
	Not Known/GISAID	–	–	1,239	1,239	
134	Delta	–	–	234	234	1,619
	Not Known/GISAID	–	–	1,385	1,385	
138	Delta	–	–	112	112	1,219
	Not Known/GISAID	–	–	1,107	1,107	
140	Delta	–	–	18,411	18,411	22,761
	Not Known/GISAID	–	–	4,350	4,350	
141	Delta	–	–	821	821	2,800
	Not Known/GISAID	–	–	1,977	1,977	
	Not Known/NCBI	–	1	1	2	
142	Delta	–	–	3,637	3,637	9,165
	Not Known/GISAID	–	–	5,528	5,528	
148	Delta	–	–	51	51	1,165
	Not Known/GISAID	–	–	1,114	1,114	
149	Delta	–	–	744	744	4,597
	Not Known/GISAID	–	–	3,853	3,853	
150	Delta	–	–	857	857	1,431
	Not Known/GISAID	–	–	574	574	
153	Delta	–	–	1,802	1,802	2,342
	Not Known/GISAID	–	1	539	540	
159	Delta	–	–	316	316	1,229
	Not Known/GISAID	–	–	913	913	
165	Delta	–	–	1,049	1,049	2,303
	Not Known/GISAID	–	–	1,254	1,254	
170	Delta	–	1	548	549	1,656
	Not Known/GISAID	–	1	1,097	1,098	
	Not Known/NCBI	–	6	3	9	
174	Delta	–	–	194	194	1,130
	Not Known/GISAID	–	–	936	936	
178	Delta	–	–	1,606	1,606	2,076
	Not Known/GISAID	–	–	470	470	
180	Delta	–	–	225	225	1,482
	Not Known/GISAID	–	–	1,257	1,257	
185	Delta	–	–	962	962	1,371
	Not Known/GISAID	–	–	409	409	
196	Delta	–	–	1,486	1,486	1,967
	Not Known/GISAID	–	–	481	481	
212	Delta	–	–	330	330	1,595
	Not Known/GISAID	–	–	1,263	1,263	
	Not Known/NCBI	–	2	–	2	
226	Delta	–	–	13	13	1,125
	Not Known/GISAID	–	–	1,112	1,112	
232	Delta	–	–	257	257	1,253
	Not Known/GISAID	–	–	996	996	
240	Delta	–	–	4,266	4,266	4,999
	Not Known/GISAID	–	–	733	733	
244	Alpha	–	–	9	9	1,226
	Delta	–	–	1,077	1,077	
	Not Known/GISAID	–	1	133	134	
	Not Known/NCBI	–	3	1	4	
	Omicron	–	–	2	2	
469	Delta	–	–	2,010	2,010	2,047
	Not Known/GISAID	–	–	37	37	
536	490R-GH	–	–	111	111	127
	Not Known/GISAID	–	–	16	16	
596	Delta	–	–	1,368	1,368	1,509
	Not Known/GISAID	–	–	133	133	
	Omicron	–	–	8	8	
616	Delta	–	–	1,230	1,230	1,232
	Omicron	–	–	2	2	
650	Omicron	–	–	814	814	814
651	Omicron	–	–	398	398	398

**Table 2:** Quarterly number of viruses in groups of similar viruses from Fig. 3

Groups by similarity	Labels	Year								Total
		2,020				2,021				
		First Quarter	Second Quarter	Third Quarter	Fourth Quarter	First Quarter	Second Quarter	Third Quarter	Fourth Quarter	
A	6	1,543	643	6	1	–	2	3	–	2,198
	9	4,631	7,189	2,390	1,557	828	284	1	–	16,880
	42	286	482	372	188	9	–	3	–	1,340
	244	1	–	–	3	27	1,064	112	19	1,226
	125	2	1	4	916	1,785	1,064	514	161	4,447
	96	46	53	32	19	29	175	742	50	1,146
B	1	1	76	2,506	6	–	–	–	–	2,589
	4	–	43	1,768	4	1	–	–	–	1,816
C	121	–	–	–	–	5	621	1,487	921	3,034
	240	–	–	–	–	6	4,281	604	108	4,999
	469	–	–	–	–	1	2,009	24	13	2,047
	140	–	–	–	–	4	18,493	1,832	2,432	22,761
	142	–	–	–	–	20	3,666	2,749	2,730	9,165
	148	–	–	–	–	–	57	692	416	1,165
	149	–	–	–	–	26	764	2,459	1,348	4,597
	159	–	–	–	–	10	333	736	150	1,229
	165	–	–	–	–	12	1,052	965	274	2,303
	174	–	–	–	–	4	227	505	394	1,130
	226	–	–	–	–	1	15	530	579	1,125
	232	–	–	–	–	–	259	712	282	1,253
	185	–	–	–	–	13	970	133	255	1,371
	196	–	–	–	–	65	1,465	283	154	1,967
	150	–	–	–	–	38	838	377	178	1,431
	138	–	–	–	–	4	112	550	553	1,219
	212	–	1	1	–	2	335	793	463	1,595
	178	–	–	–	–	–	1,608	283	185	2,076
	180	–	–	–	–	–	260	884	338	1,482
	153	1	–	–	–	–	1,813	367	161	2,342
	124	–	–	–	–	–	85	949	272	1,306
	104	19	141	92	189	132	799	2,380	1,272	5,024
	130	–	–	–	–	–	61	803	396	1,260
	170	–	–	–	8	4	593	719	332	1,656
	61	18	53	127	39	14	74	1,337	888	2,550
	596	–	–	–	–	33	1,335	131	10	1,509
D	141	1	–	–	–	290	866	1,442	201	2,800
E	616	–	–	–	–	3	1,227	–	2	1,232
F	90	62	176	111	85	56	1,191	1,323	882	3,886
G	134	–	–	–	–	–	249	952	418	1,619
H	536	–	–	–	–	–	–	–	127	127
I	650	–	–	–	–	–	–	–	814	814
	651	–	–	–	–	–	–	–	398	398

### Feature importance extraction analysis results

The random forest and shapely value (SHAP) were used to extract which part of the data was characteristic of each cluster. Here, 80% of the data was used as training data for the model while the remaining 20% was used to verify that the model was able to correctly classify the input data into appropriate clusters. In both the random forest and shapely value models, the classification accuracy for each cluster was over 99%, so it can be considered that the features extracted through the random forest or shapely value were important. Using feature importance extraction models, an attempt was made to determine which codons and amino acids played a major role in distinguishing each group. The cluster with the largest number of clusters in each group was designated as the cluster representing the group, and the differences between the clusters were examined. That is, the differences between clusters 9, 1, 125, 140, 536, and 650 were analysed through feature importance extraction models.

**Fig. 4:**
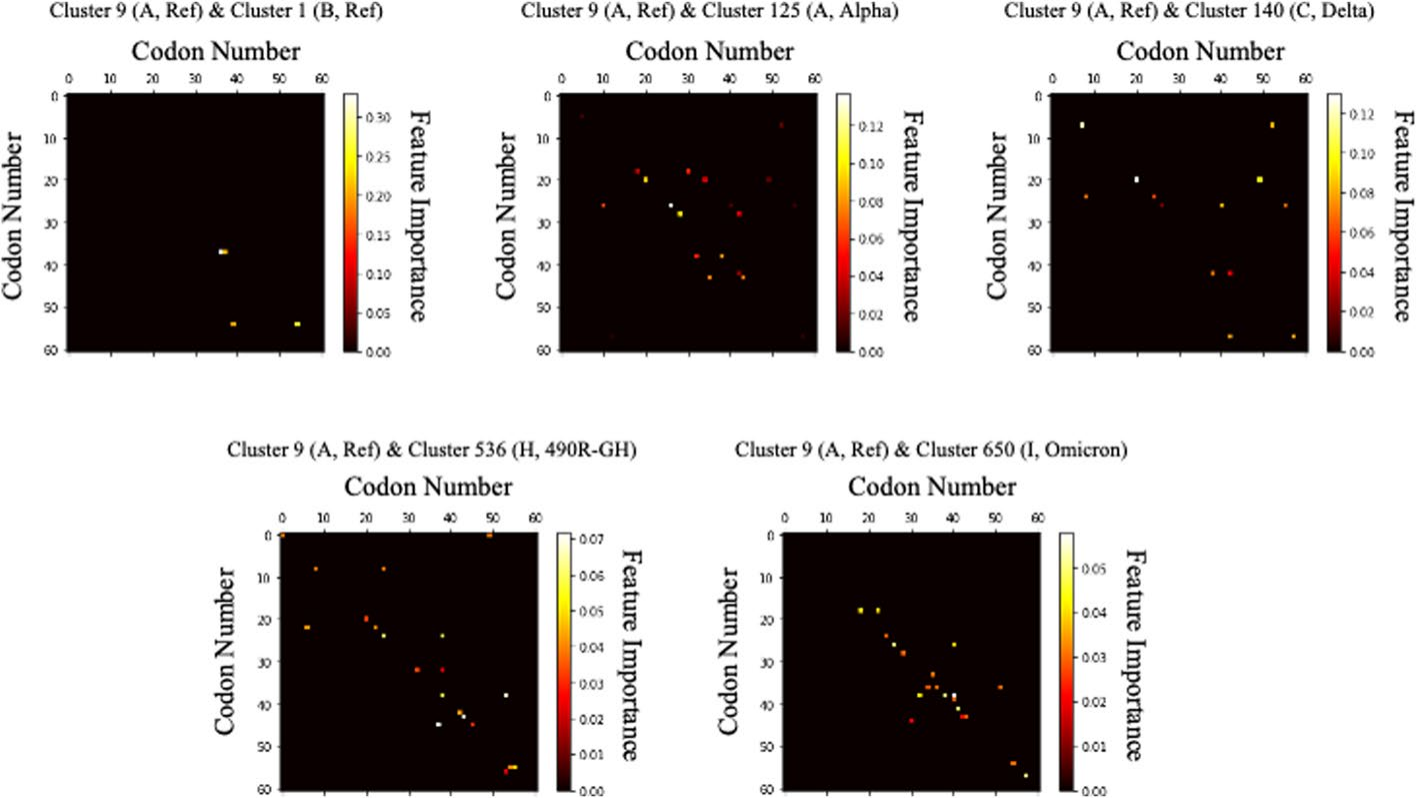
Features that played a large role in the classification of each cluster extracted through the random forest (the closer to white, the greater the role): 3,721 variables were displayed as 61 × 61 images, and features that played a major role in classifying each cluster are displayed in bright colours. It can be confirmed that only a small number of features are mutated between clusters 9 and 1, and it can be confirmed that many mutations are occurring among other clusters

The random forest results for the four different clusters are shown in Fig. 4. The heat maps shown in Fig. 4 indicated which parts of the data played a decisive role in dividing the clusters. The closer a colour is to white in the heatmap, the more important that feature is in splitting the cluster. By contrast, the closer the colour is to black, the smaller of a role that feature plays. All points were expressed as values between 1 and 0, and the sum of the 3,721 values was 1.0. The value of each point indicated the percentage of the contribution made by the corresponding feature when dividing the cluster. Each heatmap showed the features that played a big role when the corresponding column cluster and row cluster were divided.

**Fig. 5:**
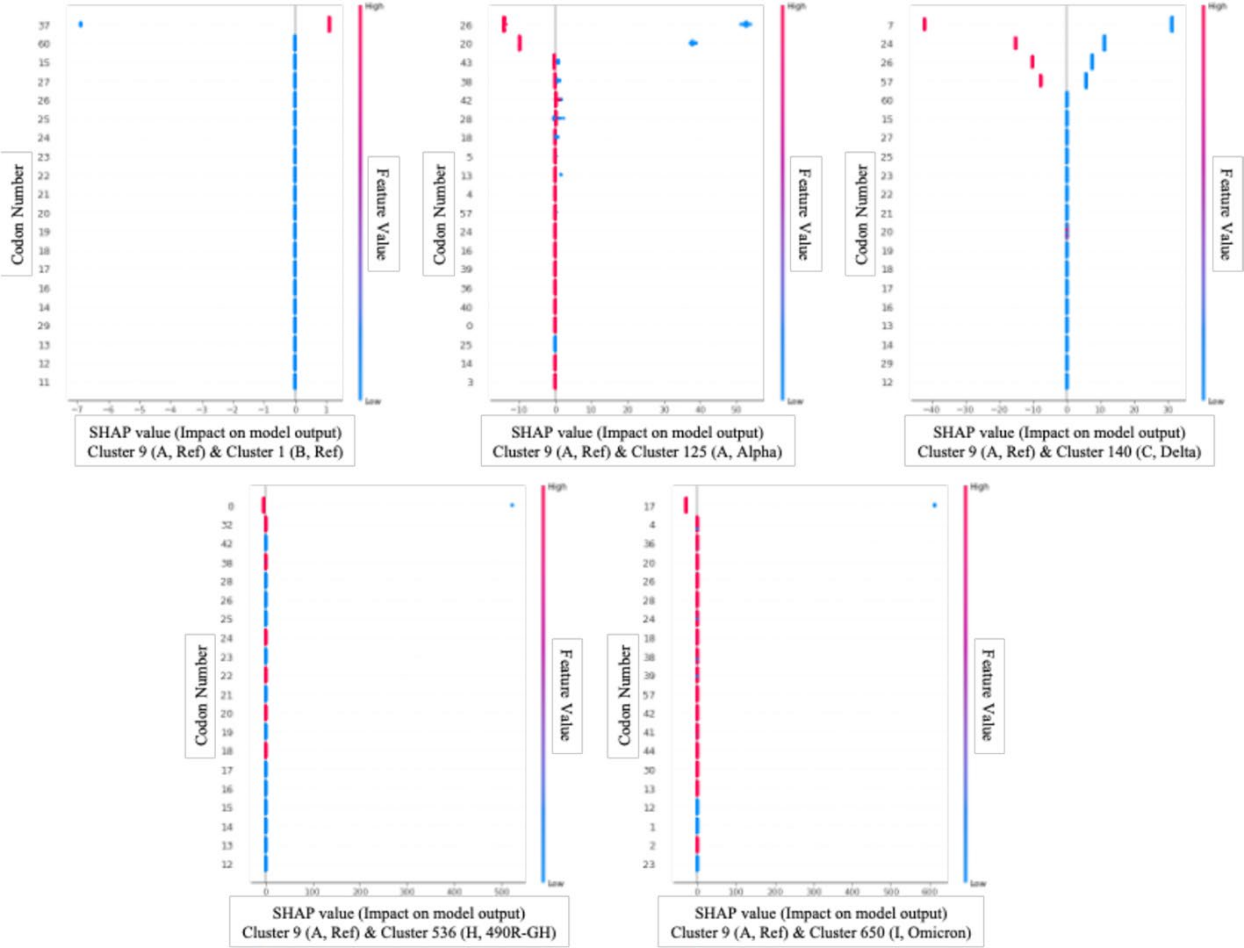
Features that played a major role in the classification of each cluster extracted through Shapely Value (SHAP): Each graph shows which codon change had a great effect on distinguishing cluster 9 from other clusters, and from the top, they are listed in order of the most influential variable among 3,721 variables. In other graphs other than the first graph comparing clusters 9 and 1, the smaller the variable value is, the closer to the 9th cluster, and the larger the variable value, the closer to the other clusters. Conversely, in the first graph, the larger the variable value, the closer it is to cluster 9, and the smaller it is, the closer it is to cluster 1

Feature importance extraction using SHAP was performed to compare the analysis results of the latest feature importance extraction technique and the classic random forest technique. The SHAP is one of the latest feature importance extraction techniques based on deep learning and is widely used recently as a tool to explain the prediction results of deep learning-based models. Figure 5 shows the result of feature importance extraction through SHAP using deep learning-based Light Gradient Boosting Method (LGBM). Figure 5 overlays feature importance and feature effects. Each point in Fig. 5 is a Shapley value and observation value for a feature. The x-axis was determined by the Shapley value and the y-axis was determined by the feature. Colour indicated the value of a feature from low to high, and as overlapping points were nested in the y-axis direction, the distribution of Shapley values per feature can be seen. Also, the features were sorted according to their importance. Each feature was indicated by a number, and each number indicated an index in Table 3. That is, each number indicated a codon matching the index in Table 3.

**Table 3:** Codons according to the index number: The codon number shown in each graph in Figs. 4 and 5 indicates the codon corresponding to the index number in the table

Index	Codon	Index	Codon	Index	Codon	Index	Codon	Index	Codon	Index	Codon
1	UUU	12	AUG	23	CCA	34	UAC	45	GAA	56	AGA
2	UUC	13	GUU	24	CCG	35	CAU	46	GAG	57	AGG
3	UUA	14	GUC	25	ACU	36	CAC	47	UGU	58	GGU
4	UUG	15	GUA	26	ACC	37	CAA	48	UGC	59	GGC
5	CUU	16	GUG	27	ACA	38	CAG	49	UGG	60	GGA
6	CUC	17	UCU	28	ACG	39	AAU	50	CGU	61	GGG
7	CUA	18	UCC	29	GCU	40	AAC	51	CGC		
8	CUG	19	UCA	30	GCC	41	AAA	52	CGA		
9	AUU	20	UCG	31	GCA	42	AAG	53	CGG		
10	AUC	21	CCU	32	GCG	43	GAU	54	AGU		
11	AUA	22	CCC	33	UAU	44	GAC	55	AGC		

We examined at the codon differences between virus 9, which belongs to cluster A and contains a reference sequence that originally appeared in Wuhan, China, and other groups using Random Forest and SHAP. Table 4 shows the feature importance extracted from each feature importance extraction model. The higher the priority value in both models, the more important the feature is. In general, deep learning-based predictive models showed better performance when a large amount of data was collected. The two models showed similar results in clusters 1, 125, and 140 with a large number of data. However, in the case of new variants, 490R-GH and Omicron, the performance of the SHAP model using deep learning-based LGBM appeared to be poor because the number of data was relatively small. Therefore, subsequent analysis was conducted based on the results of using a random forest, which showed relatively good performance even when the amount of data was small.

**Table 4:** Codons that played an important role in distinguishing clusters from other clusters from cluster 9, a reference sequence that first occurred in Wuhan, China

Cluster (group)	Codon (amino acid)	Cluster 1 (B, Ref)		Cluster 125 (A, Alpha)		Cluster 140 (C, Delta)		Cluster 536 (H, 490R-GH)		Cluster 650 (I, Omicron)	
		Random forest	SHAP	Random forest	SHAP	Random forest	SHAP	Random forest	SHAP	Random forest	SHAP
Cluster 9 (A, Ref)	AGC (SER)	0.26	0	0	0	0	0	0	0	0.03	0
	CAG (GLN)	0.21	1.915	0	0	0	0	0	0	0	0
	CCU (PRO)	0	0	0.088	15.69	0.13	0	0.032	0	0	0
	CUG (LEU)	0	0	0.012	0.018	0.12	38.2	0	0	0	0
	GGU (GLY)	0	0	0.01	0	0.08	5.627	0	0	0.05	0
	ACU (THR)	0	0	0	0	0.06	11.35	0.061	0	0.03	0
	GAU (ASP)	0	0	0.024	0.160	0.04	0	0.041	0	0	0
	ACA (THR)	0	0	0.137	22.160	0.02	8.931	0	0	0.05	0
	GAC (ASP)	0	0	0.074	0.297	0	0	0.068	0	0.03	0
	AAU (ASN)	0	0	0.08	0.181	0	0	0.058	0	0.05	0
	AGA (ARG)	0	0	0	0	0	0	0.05	0	0	0
	CCA (PRO)	0	0	0	0	0	0	0.04	0	0	0
	UUU (PHE)	0	0	0	0	0	0	0.04	7.758	0	0
	AUU (ILE)	0	0	0	0	0	0	0.038	0	0	0
	UAU (TYR)	0	0	0	0	0	0	0.032	0	0	0
	GAG (GLU)	0	0	0	0	0	0	0.03	0	0	58.34
	AAG (LYS)	0	0	0	0	0	0	0	0	0.05	0
	UCA (SER)	0	0	0.033	0.043	0	0	0	0	0.04	0
	CAA (GLN)	0	0	0	0	0	0	0	0	0.03	0
	GCU (ALA)	0	0	0.097	0.144	0	0	0	0	0.03	0

In Table 4, it can be seen that the virus in cluster 1 showed changes in Glutamine (CAG) and AGC (Serine), which was the next-most prevalent after the virus in cluster 9 was first found. The changes in ACA (threonine), GCU (alanine), CCU (proline), AAU (asparagine), GAC (aspartic acid), UCA (serine), GAU (aspartic acid), CUC (leucine), GGU (glycine) were detected in the group in which only the Alpha variant was extracted from cluster 125. Changes in CCU (proline), CUG (leucine), GGU (glycine), ACU (threonine), GAU (aspartic acid), and ACA (aspartic acid) were detected in cluster 140, which represented the Delta variation with the highest global prevalence (threonine). 490R-GH, CCU (proline), ACU (threonine), GAU (aspartic acid), GAC (aspartic acid), AAU (asparagine), AGA (arginine), CCA (proline), UUU (phenylalanine), AUU (isoleucine), UAU (tyrosine), and GAG (glutamic acid) were found in Cluster 536, the most recently discovered mutation. Lastly, changes in AGC (serine), GGU (glycine), ACU (threonine), GAC (aspartic acid), AAU (asparagine), and AAG (lysine) were observed in the Omicron mutation, which has recently become a hot topic.

## Discussion

In our study, we vectorized sequence data of the portion corresponding to the spike protein of SARS-CoV-2 from a large sample cohort around the world. By applying various machine learning techniques to this vectorized data, it provides information on how each sequence data forms a group according to the difference in sequence, and what codons are changed in viruses constituting different groups. Various characteristics appear through mutations in different codons for each mutation, and these mutations affect various characteristics such as virus transmission power and immunity. For example, mutation of important residues in the RBD of the spike protein can enhance the interaction and thus increase the ability of virus to spread [4]. Also, as described by McCallum et al., mutations in the spike protein may make the vaccine or treatment less effective [4]. Repetitive mutations in the same region are also found in several viruses, which, according to Van Dorp et al., is likely a positive selection phenomenon indicating adaptation of SARS-CoV-2 in the human host [45]. In addition, some recurrent mutations may have been induced by host immunity, showing no evidence of increased viral transmission [46]. However, a significant proportion of the detected anomalies are indicative of individual events based on what can be inferred from the available data. This indicates the need to further collect SARS-CoV-2 isolates and monitor for emerging mutants [47]. Therefore, it is very important to understand the characteristics of each virus by analyzing which codon changes were found in existing and emerging mutations.

The mutation of each codons found in various groups identified using suggesting method is compared to results of several related prior studies to verify if the experimental results of this study are reasonable. According to [4, 45], glutamine and serine both play important roles in improving ACE2 binding. It can therefore be inferred that the group B virus spread more rapidly than existing viruses at the time due to mutations in its glutamine and serine.

According to Gómez, C. E., Perdiguero, B., and Esteban, M., the Alpha variant was found to have amino acid modifications within six major residues of the receptor binding domain (RBD) [46]. Therefore, it can be inferred that the cluster 125 virus, in which the same 6 amino acid mutations were found as shown in Table 4, was the Alpha variant. In addition, in this study, threonine and serine mutations were distinguished as important features in the alpha variant, and a previous study suggested that the serine and threonine mutations found in the alpha variant enhance the local hydrogen bonding network, thereby enhancing the binding affinity for ACE2 [47]. Therefore, it can be considered that the proposed method captures the important mutations of the Alpha variant well. After the alpha variant was generated and propagated, a delta variant with very strong diffusivity was discovered.

As shown in Table 4, the CCU (proline) mutation in the Delta variant is the amino acid that played the biggest role in distinguishing the delta from the reference. A recent study related to the delta variant asserted that proline mutation played a very important role in changing the dominating variant into delta [48]. Also, the changes in CUG (leucine) and ACA (threonine), which have the next highest feature importance, were used as indicators to classify delta variants [49, 50]. It is known that a specific region of the N-terminal domain of the spike protein was vulnerable to antibody recognition and attack, and the accumulation of mutations in these antigenic supersites increased the possibility of immune escape [51]. However, since a mutation was detected in GGU (glycine) belonging to the region considered to be an antigenic supersite in the Delta variant, it was one of the most important parts of vaccine-related research [44]. This glycine mutation could also be extracted as an important feature in the results of this study. As a result, it was discovered that the suggested method correctly identified the delta variant that caused the most confirmed cases after the reference virus, as well as the codon that played a key role in delta variant differentiation.

At the end of 2021, new variants Omicron and 490R-GH were reported. Through this study, it was confirmed that both variants exhibited distinct characteristics and constitute different clusters. In Omicron, as shown in Table 4, AGC, UCA (serine) and ACU, ACA (threonine) mutations that enhance the binding affinity for ACE2 by enhancing the local hydrogen bonding network in the Alpha variant were identified. In addition, as a mutation in GGU (glycine) related to immunity was also observed, it was expected that the Omicron variant would have much higher spreading power than the existing reference virus.

In the new variant 490R-GH, as shown in Table 4, CCU, CCA (proline), which played a major role in dominating the Delta variant, and ACU (threonine), which was used to differentiate the Delta variant, were found. While many of the new mutations closely resembled the Delta variant's features, aspartic acid, asparagine, and arginine were also detected, necessitating further research into how these additional mutations may affect them.

## Conclusion

In this study, the sequence data of the SARS-CoV-2 virus was pre-processed into numerical data, vectorized, and visualized in two-dimensional space that can be more easily interpreted by humans. Data and similar viruses were clustered using the method proposed in this study. The approach compared sequence data to selected reference data, which in this example is the original virus, and then calculated the similarity between the target and reference sequences for each region, with the results expressed in a matrix form. It was feasible to vectorize the virus sequences using a variety of techniques before performing a clustering analysis because this type of data may be quantified. This made it easier to observe the occurrence of virus mutations.

One of the most noteworthy parts of this study is that each sequence data was digitized and vectorized through the proposed pre-processing method. Through digitization and vectorization of sequence data, it was possible to consider a method that actively utilizes computing power such as machine learning for numerous sequence data. By applying a machine learning technique that can handle large amounts of data at once to sequence data analysis, we were able to take advantage of several advantages over conventional methods. Existing tree-based methods have limitations in comparative analysis of large amounts of data. However, the proposed method analysed a large amount of data at once and used only computing power and several predictive models in a more objective way to distinguish variants and extract major amino acid mutations. Through the cluster analysis results, the formation of clusters between various variants can be confirmed in a two-dimensional graph, and the observed major amino acid mutations have been shown to be quite accurate through the results of recent related studies. Furthermore, it was confirmed that the Omicron and 490R-GH variants, which were discovered relatively recently and did not have a lot of data, were distinguished well. Our approach also has the advantage of being able to quickly identify which parts of the virus have mutated and allows us to easily examine the differences between mutated groups. In this study, we only focused on the nine largest groups of the mutated virus with the highest number of cases; however, we are continuously collecting data and analysing it to find new mutations. Since the proposed method can handle a wide variety of data sequences, it can be used for all kinds of diseases, including influenza and SARS-CoV-2. As such, we expected that the proposed method has the potential to become one of the most effective methods for the analysis of disease mutations.

In summary, through the proposed method, it is possible to quickly and accurately identify a virus through dimensionality reduction and clustering analysis without examining countless virus sequences one by one. In addition, as discussed in the discussion, it is not limited to simply determining which virus a virus is, but it is also possible to determine which part of the sequence in which each mutation occurs. So far, there has been no research case in which viral sequence data is vectorized and analyzed using various predictive models, including machine learning techniques, like the method proposed. Furthermore, there was no tool that could scatter a large amount of data in two dimensions to see how viruses form a community. The proposed methodology is a very original method that compares several viruses with a single reference virus and quantifies sequence data based on virus similarity, and is a methodology that serves as a cornerstone for objective analysis based on a large amount of sequence data.

Through this study, it was possible to identify which codon and amino acid mutations are important in various variants, but there was a problem that needs to be understood through other additional experiments to determine what role these parts play. Therefore, in future research, it is necessary to extract the RBD or Furin Cleavage Site, which has a great influence on the spread of the virus, or the NTD-Antigenic Supersite, which is highly related to immunity, and conduct the experiment. Furthermore, it is believed that more research is needed to determine what features exist across variations that share mutations in the same amino acid.

## Supplementary Information

**Additional file 1.** Result graphs for additional dimensionality reduction techniques, tables containing more detailed information about data, and additional information about experimental results are provided.

## Data Availability

The datasets generated and/or analysed during the current study are available in the NCBI’s SARS-CoV-2 Data Hub and GISAID, https://www.ncbi.nlm.nih.gov/sars-cov-2/, https://www.gisaid.org/, respectively. For GISAID, account registration is required. The python scripts used during the current study are available from the corresponding author on reasonable request.

## Abbreviations

SARS-CoV-2: Severe Acute Respiratory Syndrome Corona Virus 2
WHO: World Health Organization
NGS: Next Generation Sequencing
t-SNE: T-Stochastic Neighbour Embedding
DBSCAN: Density Based Spatial Clustering of Applications with Noise
NCBI: National Centre for Biotechnology Information
RBD: Receptor Binding Domain
LGBM: Light Gradient Boosting Method
SHAP: Shapely Value
