# Supplementary material for: NGS data vectorization, clustering, and finding key codons in SARS-CoV-2 variations
Source: BMC Bioinformatics. 2022 May 17;23:187. doi: 10.1186/s12859-022-04718-7 (PMC9113074; doi:10.1186/s12859-022-04718-7)
Supplement: Supplementary file 1 — Additional file 1. Result graphs for additional dimensionality reduction techniques, tables containing more detailed information about data, and additional information about experimental results are provided. [file 12859_2022_4718_MOESM1_ESM.docx]

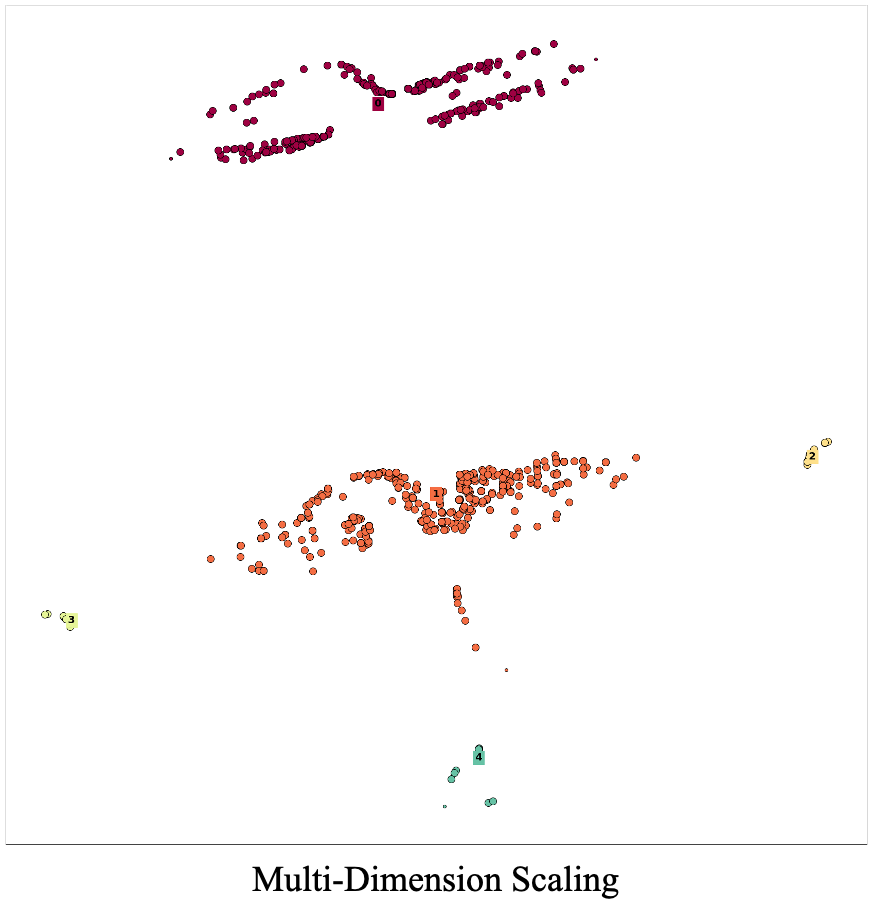

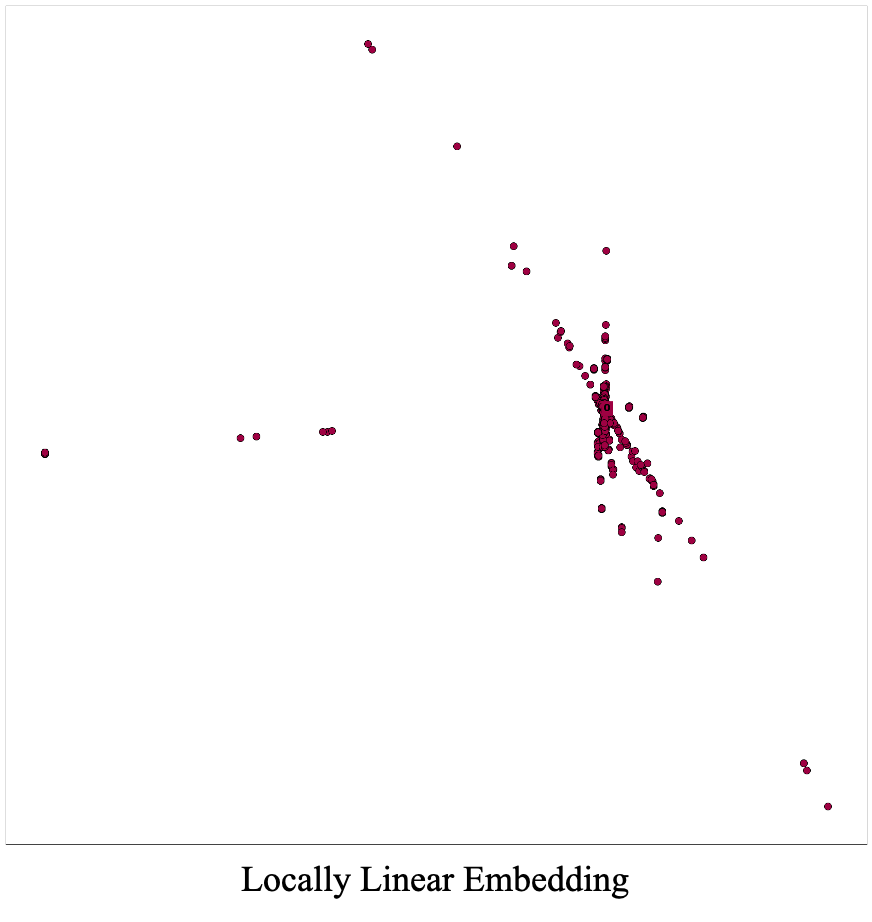


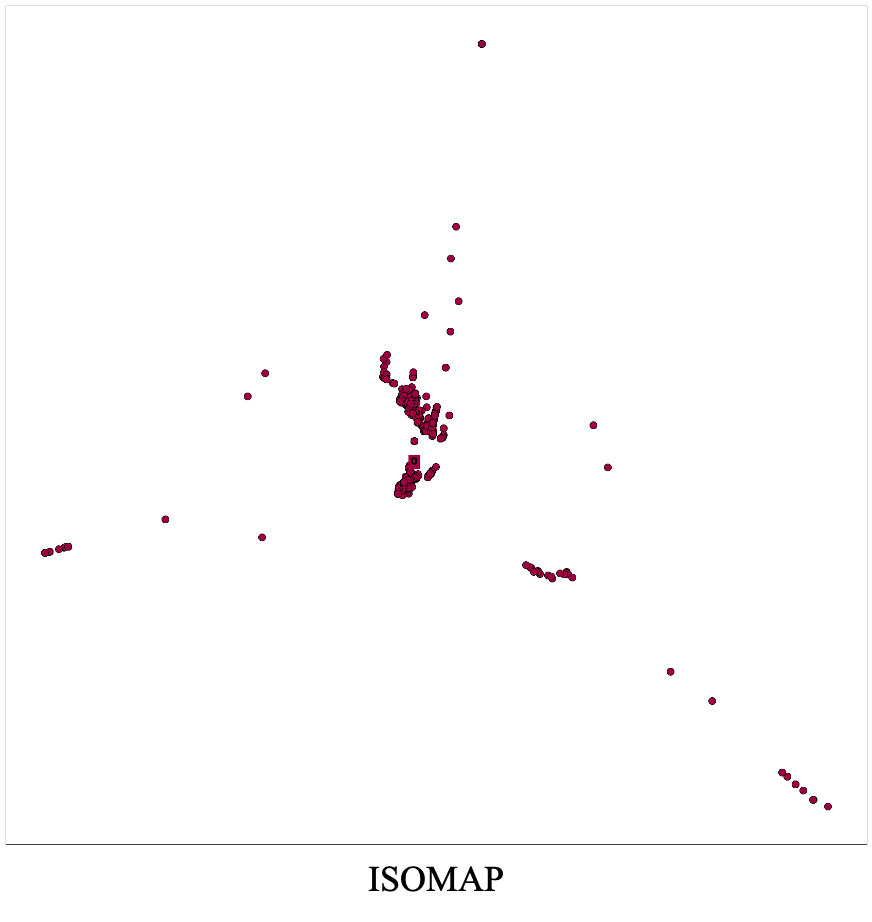

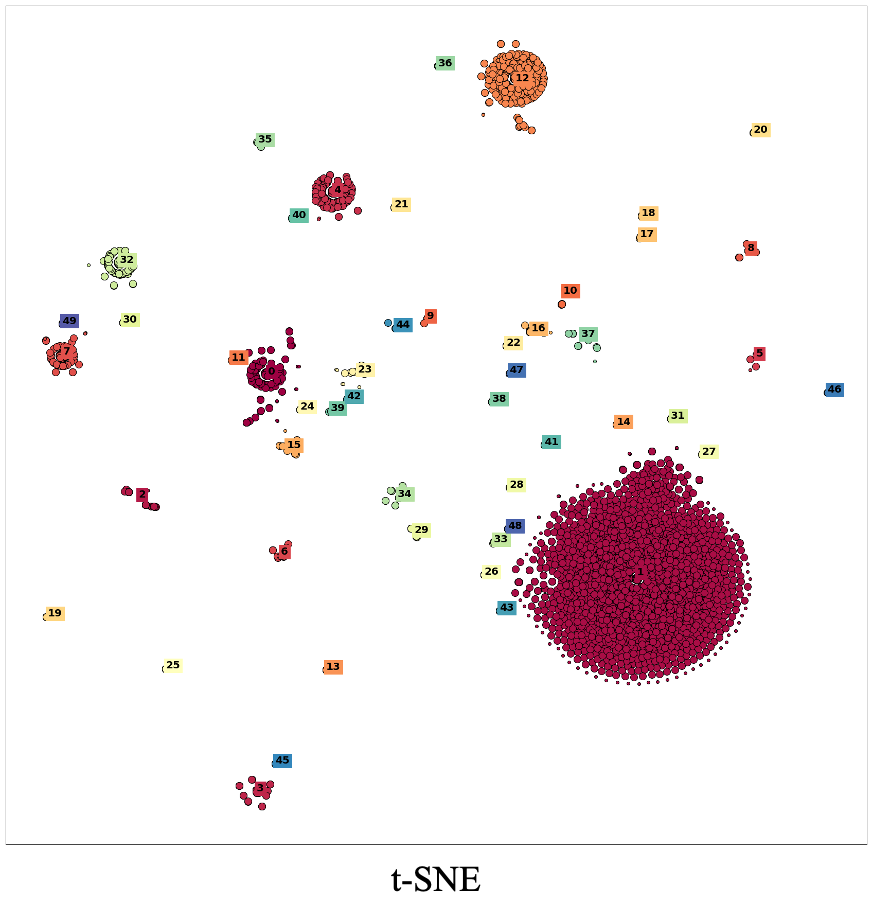


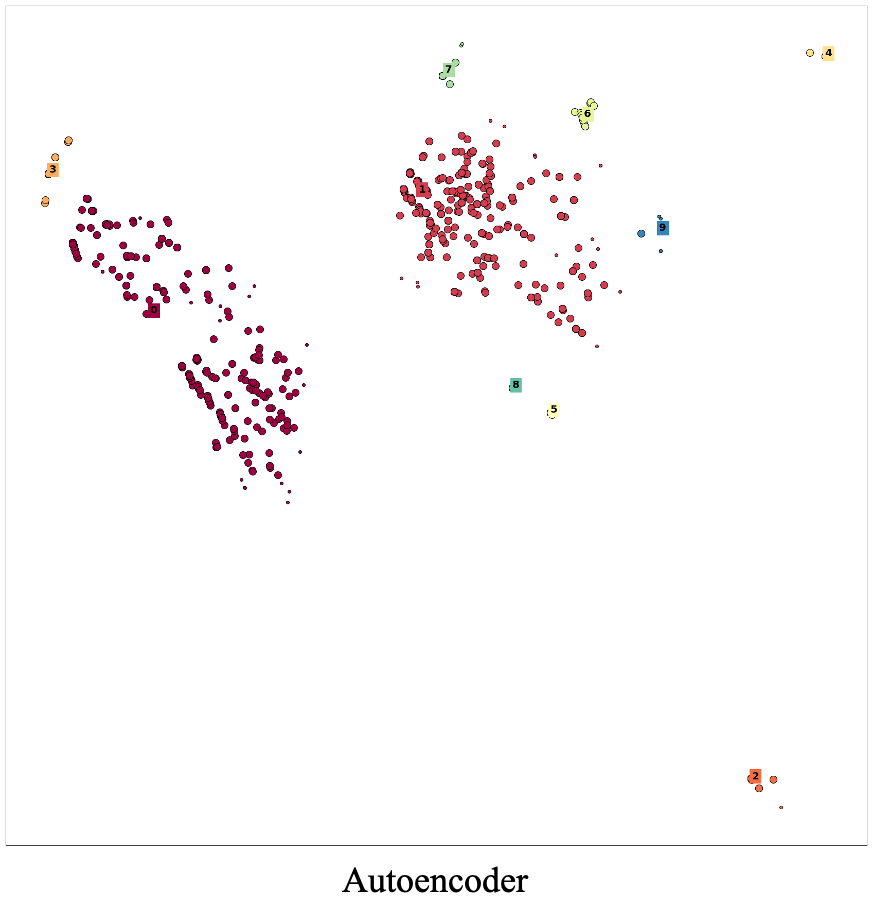


Figure S1. Dimension reduction results by applying MDS, LLE, ISOMAP, t-SNE, and Autoencoder by extracting 5,000 random samples from the entire collected data

Table S1. Number of data samples collected by region: Data from Asia, Africa, North America, South America, and Oceania, which are six continents except Antarctica, where no confirmed cases have occurred, were collected and used for analysis.

| Continent | Sub Region | Year | | | | Total |
| --- | --- | --- | --- | --- | --- | --- |
|  |  | 2019 | 2020 | 2021 | Total |  |
| Africa | Eastern Africa | 0 | 18 | 524 | 542 | 3,606 |
|  | Northern Africa | 0 | 368 | 578 | 946 |  |
|  | Southern Africa | 0 | 1 | 1,403 | 1,404 |  |
|  | Western Africa | 0 | 67 | 637 | 704 |  |
|  | Middle Africa | 0 | 0 | 10 | 10 |  |
| America | Central America and Caribbean | 0 | 157 | 16,181 | 16,338 | 65,382 |
|  | North America | 0 | 26,919 | 16,414 | 43,333 |  |
|  | Temperate South America | 0 | 27 | 1,207 | 1,234 |  |
|  | Tropical South America | 0 | 276 | 4,201 | 4,477 |  |
| Asia | Central Asia | 0 | 4 | 44 | 48 | 28,778 |
|  | Eastern Asia | 15 | 2,817 | 4,318 | 7,150 |  |
|  | Southern Asia | 0 | 1,231 | 8,448 | 9,679 |  |
|  | South-East Asia | 0 | 132 | 10,751 | 10,883 |  |
|  | Western Asia | 0 | 379 | 639 | 1,018 |  |
| Europe | Eastern Europe | 0 | 659 | 12,911 | 13,570 | 115,356 |
|  | Northern Europe | 0 | 809 | 61,390 | 62,199 |  |
|  | South West Europe | 0 | 698 | 38,889 | 39,587 |  |
| Oceania | Oceania | 0 | 10,394 | 557 | 10,951 | 10,951 |
| Total | | | | | | 224,073 |

Table S2. Number of data samples collected by variant name: The reference virus first discovered in Wuhan, China in 2019, Alpha, Beta, and Gamma mutations discovered in the second half of 2020, Delta mutations found in the first half of 2021, Omicron mutations and 490R-GH mutations found in the second half of 2021 were collected.

| Variant | Year | | | Total |
| --- | --- | --- | --- | --- |
|  | 2019 | 2020 | 2021 |  |
| B.1.1.7 (Alpha) | 0 | 1,075 | 1,955 | 3,030 |
| B.1.351 (Beta) | 0 | 99 | 852 | 951 |
| P.1 (Gamma) | 0 | 83 | 490 | 573 |
| B.1.617 (Delta) | 0 | 12 | 72,026 | 72,038 |
| B.1.1.529 (Omicron) | 0 | 0 | 3,332 | 3,332 |
| B.1.640 (490R-GH) | 0 | 0 | 115 | 115 |
| Unmuated or variant not identified | 15 | 43,687 | 100,332 | 144,034 |
| Total | | | | 224,073 |


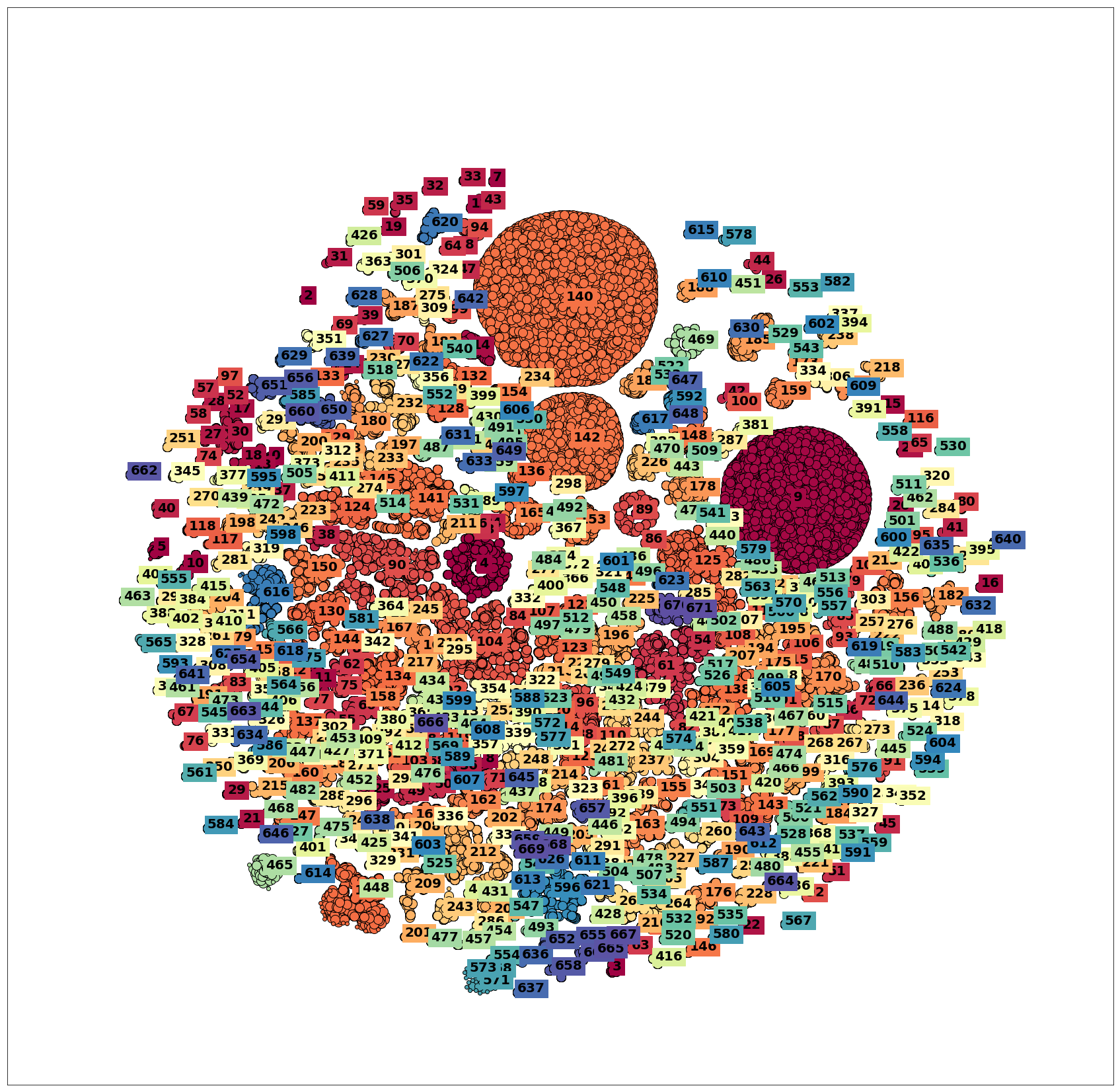


Figure S2. Result of dimensionality reduction and clustering analysis by applying t-SNE and DBSCAN to all collected data

As shown in Groups A, B, C, and I, the differences between viruses that have been labelled with the same variant were examined but were divided into multiple clusters. First, clusters 6, 9, and 42 belonging to group A only showed changes in (aspartic acid (GAU), as presented in Table S3. Clusters 1 and 4 belonging to group B showed a similarity of 0.99999 in the virus similarity analysis shown in Figure 3. Therefore, it can be said that the viruses from both clusters were in fact the same type of virus. In the case of group C consisting of delta variants, since too many sub mutations were included together, four representative clusters containing more than 4,000 data were selected for comparative analysis. As shown in Table S4, among the clusters belonging to group D, 140, 142, 149, and 240 were compared, and changes in four different codons were confirmed. Changes were commonly found in ACU (threonine), GGU (glycine), CCU (proline), and GAU (aspartic acid). Finally, table S5 shows the difference between clusters 650 and 651 in group I representing the Omicron variants. Even though they were both labeled with the identical Omicron variation, it was established that the two clusters differed in 9 codons and in many parts.

Table S3. Features that played an important role in the division of each cluster in group A

| Group A | Codon (Amino Acid) | Cluster 6 | Cluster 9 | Cluster 42 |
| --- | --- | --- | --- | --- |
| Cluster 6 | GAU (ASP) | 0 | 0.521 | 0.520 |
| Cluster 9 | GAU (ASP) | 0.521 | 0 | 0.480 |
| Cluster 42 | GAU (ASP) | 0.520 | 0.480 | 0 |

Table S4. Features that played an important role in the division of each cluster in group C

| Cluster | Codon (Amino Acid) | Cluster 140 | Cluster 142 | Cluster 149 | Cluster 240 |
| --- | --- | --- | --- | --- | --- |
| Cluster 140 | ACU (THR) | 0 | 0.441 | 0.212 | 0.120 |
|  | GGU (GLY) | 0 | 0 | 0.223 | 0.121 |
|  | CCU (PRO) | 0 | 0 | 0 | 0.130 |
|  | GAU (ASP) | 0 | 0 | 0 | 0.100 |
| Cluster 142 | ACU (THR) | 0.441 | 0 | 0 | 0 |
|  | GGU (GLY) | 0 | 0 | 0.308 | 0.191 |
|  | CCU (PRO) | 0 | 0 | 0 | 0.120 |
|  | GAU (ASP) | 0 | 0 | 0 | 0.071 |
| Cluster 149 | ACU (THR) | 0.212 | 0 | 0 | 0 |
|  | GGU (GLY) | 0.223 | 0.308 | 0 | 0 |
|  | CCU (PRO) | 0 | 0 | 0 | 0.210 |
|  | GAU (ASP) | 0 | 0 | 0 | 0.170 |
| Cluster 240 | ACU (THR) | 0.120 | 0 | 0 | 0 |
|  | GGU (GLY) | 0.121 | 0.191 | 0 | 0 |
|  | CCU (PRO) | 0.130 | 0.120 | 0.210 | 0 |
|  | GAU (ASP) | 0.100 | 0.071 | 0.170 | 0 |

Table S5. Features that played an important role in the division of each cluster in group I

| Cluster | Codon (Amino Acid) | Cluster 651 |
| --- | --- | --- |
| Cluster 650 | GGU (GLY) | 0.060 |
|  | AAG (LYS) | 0.048 |
|  | GAU (ASP) | 0.046 |
|  | AAU (ASN) | 0.038 |
|  | ACA (THR) | 0.037 |
|  | CAA (GLN) | 0.027 |
|  | CUU (LEU) | 0.026 |
|  | ACU (THR) | 0.024 |
|  | GCU (ALA) | 0.024 |

In addition, the viruses of groups D, E, F and G that were tagged with the Delta variant but did not belong to group C were compared with cluster 140, which occurred the most in group C. As can be seen in Table S6, unlike the clusters in group C, which showed no significant changes other than the ACU (threonine), GGU (Glycine), CCU (Proline), and GAU (Aspartic Acid) codons, a total of 9 codons in the 141 cluster showed a different form from the 140 cluster, and 15 codons in cluster 616 as well as 8 and 9 codons in 90 cluster and 134 cluster respectively were different, showing a significant difference from cluster 140 representing group C.

Table S6. Features that played an important role in the division of clusters between 140 and 141, 616, 90, and 134

| Cluster | Codon (Amino Acid) | Cluster 141 | Cluster 616 | Cluster 90 | Cluster 134 |
| --- | --- | --- | --- | --- | --- |
| Cluster 140 | GGU (GLY) | 0.112 | 0 | 0.058 | 0.053 |
|  | ACU (THR) | 0.046 | 0 | 0.076 | 0.171 |
|  | AGG (ARG) | 0.046 | 0 | 0 | 0 |
|  | CAU (HIS) | 0.033 | 0.021 | 0 | 0 |
|  | GAU (ASP) | 0.030 | 0 | 0.027 | 0.058 |
|  | GUU (VAL) | 0.029 | 0 | 0 | 0 |
|  | AAG (LYS) | 0.027 | 0 | 0 | 0 |
|  | ACA (THR) | 0.025 | 0 | 0.012 | 0.005 |
|  | AAU (ASN) | 0.024 | 0.030 | 0 | 0 |
|  | UCG (SER) | 0 | 0.040 | 0 | 0.182 |
|  | UUC (SER) | 0 | 0.040 | 0 | 0 |
|  | GAA (GLU) | 0 | 0.040 | 0 | 0 |
|  | CCA (PRO) | 0 | 0.040 | 0 | 0 |
|  | CUU (LEU) | 0 | 0.031 | 0 | 0 |
|  | GAC (ASP) | 0 | 0.021 | 0 | 0 |
|  | CUA (LEU) | 0 | 0.021 | 0 | 0 |
|  | UUU (PHE) | 0 | 0.020 | 0 | 0 |
|  | ACC (THR) | 0 | 0.020 | 0 | 0 |
|  | GCG (ALA) | 0 | 0.020 | 0.130 | 0 |
|  | GCA (ALA) | 0 | 0.020 | 0 | 0 |
|  | GUC (VAL) | 0 | 0.020 | 0 | 0 |
|  | GUA (VAL) | 0 | 0 | 0.129 | 0.012 |
|  | CCU (PRO) | 0 | 0 | 0.019 | 0.012 |
|  | GCU (ALA) | 0 | 0 | 0.008 | 0.004 |
|  | AAC (ASN) | 0 | 0 | 0 | 0.004 |
